# An adapted model of cost-related medication nonadherence among older adult patients with chronic diseases: an Iranian qualitative study

**DOI:** 10.1186/s12877-023-03907-0

**Published:** 2023-04-01

**Authors:** Soheila Rezaei, Mohammad Peikanpour, Leila Zarei, Ghader Mohammadnezhad, Jamshid Salamzadeh

**Affiliations:** 1 Department of Pharmacoeconomics and Pharma Management, School of Pharmacy, Shahid Beheshti University of Medical Sciences, Tehran, Iran; 2 Health Policy Research Center, Institute of Health, Shiraz University of Medical Sciences, Shiraz, Iran; 3 Department of Clinical Pharmacy, School of Pharmacy, Shahid Beheshti University of Medical Sciences Sciences, Niayesh Highway, Valiasr Ave, P.O. Box 14155-6153, Tehran, Iran

**Keywords:** Chronic disease, Iran, Medications, Non-adherence, Older adults

## Abstract

**Background:**

Following the rapid aging of population, some concerns have emerged regarding increasing demand for health care services and the consequent increase in health costs. Besides, older adult patients with chronic disease are more prone to show cost-related medication non-adherence (CRN) to cope with their medication costs. The objective of this qualitative study was to develop an adopted conceptual framework on the contextual determinants that affect the CRN in older adult patients with chronic diseases.

**Methods:**

Problem-centered, semi-structured, in-depth, and face-to-face interviews, were conducted with healthcare informants in Iran, from Sep. 2021 to Feb. 2022. Collected data were analyzed using deductive and inductive analytic approaches and content analysis methodology was used to develop the model. This study applies to the COREQ checklist.

**Results:**

Fifteen informants, including 8 (60%) males, with mean ± SD age of 44.4 ± 9.7 years, entered into the study. Based on the data analysis performed on the information obtained from the interview with 3 subgroups of geriatricians, health policymakers, and pharmacists, six major themes identified as determinants: 1) socio-economic factors, 2) health system-related factors, 3) healthcare provider-related factors, 4) medication-related factors, 5) disease-related factors, and 6) patient-related factors. There were also 23 minor themes which were matched with the aforementioned six major themes.

**Conclusion:**

The final framework obtained in this qualitative study depicts CRN as an issue that is highly likely affected by six main determinants among older patients with chronic disease. Our findings emphasize that policymakers would focus on certain major themes and allocate resources to programs to improve medication therapy management in older adult patients.

**Supplementary Information:**

The online version contains supplementary material available at 10.1186/s12877-023-03907-0.

## Background

Globally, public health and medical technology improvements lead to increased life expectancy [1], leading to a growing number of older people (aged 60 years and above). By 2030, it was estimated that the older adult population will increase to 1.4 billion worldwide, two-thirds of whom will live in low- and middle-income countries [2]. Iran, as a middle-income country, is facing a significant demographic shift and massive population aging. It has been forecasted that older adult patients will rise to 10.5% in 2025 and 21.7% in 2050 in Iran, compared to 5.2% in 2000 [3].

Following the rapid demographic change, some concerns, such as increasing demand for health care services by older adult patients and the consequent significant increase in health costs, have emerged [4]. Additionally, pharmaceutical expenditure has grown significantly faster than other health expenditure categories in recent decades [5]. On the other hand, older adult patients are more likely to suffer from one or more chronic diseases. The presence of multiple chronic diseases and the need for long-term use of medications have caused an economic burden on older adults and the healthcare system [4]. As a result, it was frequently reported that many older adult patients present some non-adherent and cost-reduction behaviors to cope with their medication costs [6–10].

Cost-related medication non-adherence (CRN) is defined as insufficient use of medications because of cost. It is generally agreed individuals are subject to CRN when they show one of the following behaviors during the past 3 or 12 months: (1) not filling or delay in filling a prescription to save money, (2) skipping doses or taking less medication than prescribed or splitting pills to make medicines last longer, (3) asking a doctor to prescribe lower-cost and affordable medication/s [6, 8, 9, 11, 12].

Based on previous reports, the prevalence of CRN is around 10 percent [13]. Existing research shows that the CRN has a devastating effect on the health status of older adult patients; for example, health deterioration, an increase in the use of other medications and health care services, health costs, and higher levels of emergency department admissions [10, 14, 15]. Lee et al. (2018) reported that CRN could be influenced by multiple factors (*e.g*., disease type, disease duration, poor physical access, medication unaffordability), which might explain the broad range of CRN rates observed among older adults. The patient's attitudes and beliefs can mediate the effects of financial resource availability on CRN [13].

Even though out-of-pocket payment is a cornerstone in determining medication costs and patient adherence behavior, prior research has concluded that other non-cost-related factors might change the individual’s response to financial pressure [12]. So far, few conceptual frameworks have been developed to help understand CRN behavior and to examine the effect of contextual factors on CRN in different populations [15]. There are numerous studies reporting factors related to medication adherence in the older adults with chronic diseases [13, 16–21]. Also, there are limited studies investigating CRN in older adults with particular diseases [13, 22], nonetheless, there is a general dearth in literature on describing a comprehensive model regarding CRN behavior in older adult patients, particularly in developing countries.The objective of this study was to develop an adopted conceptual framework using opinions retrieval from healthcare informants on the contextual determinants that affect CRN in older adult populations with chronic diseases. The findings of this study may inform policymakers to identify the most important areas to mitigate the risk of CRN among older adult patients.

## Methods

### Study design, setting, and sampling

To develop an adopted model for CRN in the Iranian older adults with chronic disease, first, an in-depth literature review was conducted to identify related theoretical studies. Then, based on results of similar studies from different contexts and settings, the main hypothesis was created for the model. Finally, the proposed existing model by Piette and colleagues as well as the WHO report [15, 23], were used as a theoretical framework, so that the extracted minor themes can place and fit with existing framework.

The COREQ (COnsolidated criteria for REporting Qualitative research) checklist [24] was followed to improve the quality and transparency of the current study (see Additional file 1). This qualitative study, conducted from September 2021 to February 2022, aimed to evaluate informants’ opinions and experiences regarding the contextual determinants that may potentially impact the CRN behavior of older adult patients.

The clustering sampling method was chosen to select three sub-groups of informants a) geriatricians, b) health policymakers, and 3) pharmacists. A purposive and convenience sampling strategy was used to select eligible interviewees so that the authors were assured each sub-group provided rich data with high accuracy and reliability. Interviewees were asked to enter a semi-structured, in-depth, and face-to-face interview.

Informants were eligible to be included in the study if they were at least 35 years old, had at least ten years of related experience, and were available to be interviewed in Tehran, Iran. Interviewees were excluded if they were outside Tehran and did not have enough time or willingness to interview.

To improve the validity and reliability of this qualitative study, the four criteria of credibility, dependability, confirmability, and transformability were applied [25, 26]. Long-term engagement of researchers with the topic, and continuous comparison of the data assure credibility. In order to improve dependability, the audit process was conducted through the members of the research team. To reach confirmability, the process of data coding was reviewed. To reach transformability, the detailed explanation of the study methodology, the participants’ characteristics, and clarification of data collection and data analysis process could enable the readers to assess whether the findings are transferable to other settings or populations. Throughout this process, to test the fitness of obtained framework, continuous refinement and amendment were conducted by peer discussion, constant comparative analysis, and consideration of the current state of research.

### Data collection

A problem-centered and structured interview guide [27] was mutually developed by the research team in order to achieve structured feedback from participants. The interview guide included open-ended questions on informants’ experience with CRN behavior among older adult patients (see Additional file 2). It was developed using existing related literature [7–9] and pre-tested with three informants to ensure clarity and appropriateness of interview length. Whenever necessary, follow-up and affirmative questions were asked during the interview.

Demographic data of informants was also recorded. All interviews were conducted by the principal investigator, who is trained and experienced in qualitative research.

The interviews were privately conducted at the office or workplace of the informants. The interviews were audio-recorded. The audio recordings were converted to transcripts as soon as after the interviews. The focal points mentioned by interviewees were double-checked and confirmed verbally by the participants at the end of each interview. The interviews were not limited to a pre-planned sample size, and the interviews were continued until reaching a saturation level of the key themes. No more interviews took place when no new theme emerged during the interviews. Data saturation was obtained after 15 interviews.

### Data analysis

A combination of the deductive-inductive approach and the Framework Method for the qualitative data [28] was applied to analyze the information obtained from interviews. This qualitative data was used to categorize themes and organize them in the existing framework by content analysis methodology [15, 23]. In this regard, first, all audio recordings were converted to transcripts. The most meaningful units of text, which were considered as units of analysis, were extracted and labeled as initial codes. The extracted initial codes were reviewed and refined to obtain minor themes. In the next step, the minor themes were matched to the main determinants of CRN in older adult patients. This process was conducted by two of the researchers separately to improve the consistency of the qualitative data analysis. The MAXQDA10 program was applied to analyze the qualitative data.

## Results

In this study, 15 informants were interviewed. Table 1 shows the characteristics of the interviewees. The mean ± SD age of participants was 44.4 ± 9.7 years, and 8 (60%) of them were male. The sampling strategy was conducted to recruit three stratified subgroups of geriatricians, health policymakers, and pharmacists. Interviews lasted from 25 to 45 min (mean ± SD = 35 ± 10 min).

**Table 1:** Demographic characteristics of participants

Informants	Participant Category	Age	Gender	Educational Level
Informant 1	Geriatrician	43	Male	M.D
Informant 2		47	Male	M.D
Informant 3		44	Female	M.D
Informant 4		38	Female	M.D
Informant 5	Health policymakers	51	Female	M.D
Informant 6		61	Male	M.D
Informant 7		56	Male	M.D
Informant 8		41	Male	Ph.D
Informant 9		43	Male	M.D
Informant 10		52	Male	Pharm.D
Informant 11	Pharmacists	36	Male	Pharm.D, Ph.D
Informant 12		37	Female	Pharm.D, Ph.D
Informant 13		46	Female	Pharm.D, Ph.D
Informant 14		35	Female	Pharm.D, Ph.D
Informant 15		36	Male	Pharm.D, Ph.D

**Fig. 1:**
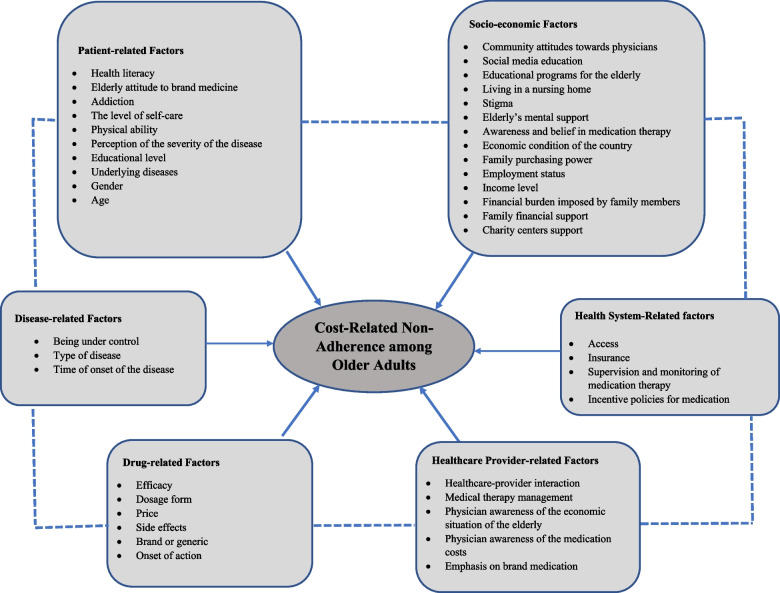
An adopted model for cost-related medication non-adherence among older adults

The results of the informants’ opinions’ retrieval are shown in Fig. 1.

The minor themes were matched with the model proposed by Piette and colleagues as well as the WHO report [15, 23], allowing to find the most appropriate theme for each of extracted minor themes. As a result, the 23 minor themes were matched with six major themes, as shown in Table 2. The extracted major themes were as follows: *Socio-economic Factors, Health System-Related Factors, Healthcare Provider-related Factors, Drug-related Factors, Disease-related Factors, and Patient -related Factors*. All major themes are subject to financial and non-financial determinants of CRN in older adult patients. Exemplary quotes which correspond to the themes can be found in Additional file 3.

**Table 2:** Major and minor themes from thematic analysis

Major Themes	Minor Themes	Main Category
Socio-economic Factors	Cultural Factors	• Community attitudes towards physicians • Social media education • Educational programs for the older adults • Living in a nursing home • Stigma • Older adult’s mental support • Awareness and belief in medication therapy
	Economic Factors	• The economic condition of the country • Family purchasing power • Employment status • Income level • Financial burden imposed by family members • Family financial support • Charity centers support
Health System-Related factors	Access	• Medicine shortage
	Insurance	• Out-of-pocket costs • Insurance coverage
	Supervision and monitoring of medication therapy	-
	Incentive policies for medication therapy	-
Healthcare Provider-related Factors	Healthcare-provider interaction	• Devoted time for the visit • Communication with the older adults • Talking about the importance of taking medicine
	Medical therapy management	• Polypharmacy management • Medication reconciliation • Teaching about medication efficacy • Management of medication side effects
	Physician awareness of the economic situation of the older adults	-
	Physician awareness of the medication costs	-
	Emphasis on brand medication	-
Drug-related Factors	Efficacy	
	Dosage form	
	Price	
	Side effects	
	Brand or generic	
	Onset of action	
Disease-related Factors	Being under control	
	Type of Disease	
	Time of onset of the disease	
Patient-related Factors	Health literacy	
	Older adults’ attitude to brand medicine	
	Addiction	
	The level of self-care	
	Physical ability	Dependency on others to supply or consume medicines
	Perception of the severity of the disease	
	Educational level	
	Underlying diseases	• Depression • Cognitive disorders
	Gender	
	Age	

### Socio-economic factors

This major theme will be described in two minor themes: cultural and economic factors.

Cultural factors refer to some determinants which exist in the nature of each society. These determinants include perceptions and behaviors observed in a society or a specific population and learned by individuals from other people around them. Certainly, these types of factors vary amongst diverse contexts. Informants emphasize that older adults can learn from society. This learning begins from childhood, finally forms their behavior, and determines the older adult's response to medication costs.

*"The knowledge and the things they get from others, from their community, or a peer, for example, about the availability of better medication, are more important, and generally the feedback they get from others is more important."* [Informant 3]

Some informants have voiced that the presentation of special learning programs in media can boost older adult’s awareness about their medical condition as follows:

*"See, the thing is, even the English websites you search on over there, there is a lot of patient education about medications, their side effects, and the progression of the disease."* [Informant 11]

Some described that cultural factors are related to family members and society supporting older adult patients.

*"Patients residing in a sanatorium can be the toughest to care for. Many of these people feel unloved, which makes them feel hapless. We live for attention, but they don't understand how to give or receive attention. It doesn't matter from whom you get attention. It can be from your older adult roommate, but it doesn't happen in a nursing home. Sometimes the caregivers there get tired of their patients. "* [Informant 1]

Another point voiced by informants is community attitudes toward physicians:

*"When there's a bad rap against doctors in the community, the older patients who distrust practitioners go and get their prescriptions, figure out the cost in their heads, do the math on paper, and say "it's not clear at all what you the doctor said."* [Informant 2]

More exemplary quotes can be found in Additional file 3.

As mentioned by all participants, economic factors were described as determinants of CRN in older adult patients. Regarding the economic condition, one of the informants declared:

*"When the economy sucks, and there isn't any job that influences the older adult's behavior toward preparing their medications. The older adult's economic situation is important, but society's economy is also important. Health influences the economy, and the economy influences health. But here, the economy as a whole affects people's medical behavior as much as any other."* [Informant 4]

Almost all of the informants claimed that the family's purchasing power could considerably impact CRN. When purchasing power decreases, older adult patients cannot even afford basic needs, let alone the cost of medications. In this regard, one of the participants said:

*"Unfortunately, there are some seniors who can't afford medicine. There are poor people in the region who can barely pay for their food, let alone their medicine. There are so many more basic needs that must be met."* [Informant 1]

In addition, being financially supported by family members can potentially persuade the older adult to take their medication. On this topic, an informant stated:

*"But those intimate relationships with the families do help; they can alleviate some of the costs. Children can cover the costs. Those with special children who care for them usually don't have a problem. The child takes charge and says, 'Don't worry, I'll find a way to get it no matter what.' But those who have to shell out cash themselves have to squeeze things in their life."* [Informant 15]

### Health system-related factors

Among health system-related factors, insurance is most emphasized by informants. Whether the older adult patient has insurance coverage or not and the level of coverage that determines the amount of out-of-pocket payments both can greatly impact the older adult's medication behavior and response to medication costs. An informant specified:

*" I have seen older adult patients not care how much a certain medication costs. They don't buy medicine that isn't covered by insurance because they feel they're paying for it all by themselves."* [Informant 5]

Another participant stated:

*"Some insurance companies even cover brand drugs. Some patients are more satisfied with that and ask their doctors to prescribe the brand drug because they're not concerned with the cost anymore, and they're not going to pay for it."* [Informant 12]

Another factor indicated by some informants is the supervision and monitoring of medication therapy.

*"Some services should be actively provided to the older adult. For example, we shouldn't say, 'Go and get medicine or do a test.' What if the patient doesn't follow? That's not an active healthcare system. In an active health care system, we need to follow up and offer services to the patient."* [Informant 2]

Many informants emphasized the effect of access and shortage of medication on CRN. The high price of these medications in the free market may also encourage the older adult not to buy and use their medications. Sometimes the preparation of medications becomes so difficult that the older adult completely give up the treatment process:

*"Having access to medicine can also be an issue. Some medications can only be found in certain pharmacies in big cities, so if you don't live nearby, it might take a while and can be cost-consuming to get there and back if you need to buy medicine."* [Informant 13]

### Healthcare provider-related factors

Among healthcare provider-related factors emerged, nearly all of the informants believed that if the interaction between the healthcare provider and the older adult is established correctly, it will be easier for the older adult to accept the physician's instructions:

*"… there is no communication between the doctor and the patients. In other words, in the communication between doctor and patient, the more information the patient gets, the more they will follow the doctor’s recommendation."* [Informant 14]

Since older adult patients take many medications because of concurrent co-morbidities and the chronic nature of diseases they have, medication therapy management has a key role in CRN behavior in older adult patients:

*"Reducing the number of medications, a patient takes can effectively save money. Imagine a diabetic whose sleep is a wreck and who gets neuropathic pain. Gabapentin helps, lowering the need for extra medications. The more we can use it and other meds like it, the higher the adherence and the lower the cost."* [Informant 4]

In this regard, some informants indicated that medication reconciliation could potentially help physicians to have better control over medical therapy management as follows:

*"In order to have a comprehensive view of medication, the doctor tells the older adult to bring all their medications with them to review all medications and prescribe the same medication that is needed for the older adult."* [Informant 10]

Additionally, informants mentioned physician’s awareness of the economic situation as well as medication costs as healthcare provider-related factors which have a potential impact on CRN among older adult patients:

*"I'm a pharmacotherapist, so this (medication cost) is a very high priority for me. I discuss this with the older adult patient. If I see that they can't afford it, I will find an alternative medication." *[Informant 13]

### Drug-related factors

Concerning the major theme of drug-related factors which affect CRN, minor themes emerged from the analysis, such as the dosage form of the drug:

*"When I order a sustained release formulation for older patients, and I explain that it's expensive, but it has not some adverse events, and it makes you better, then he/she is convinced."* [Informant 1]

All informants claimed that the price of medication directly affects CRN behavior.

In this regard, one of the participants noted:

*"Often, I find them (the older adult) asking about the price of the medication and leaving out the most expensive one, which is usually the main drug. Without it, the illness could get worse. At that time, I instruct them never to leave this out. Most of them don't accept what I say."* [Informant 15]

Side effects of medication used by older adult patients were mentioned as a drug-related factor that can be a determinant of CRN:

*"Because of a medicine side effect or even a complication caused by the illness while under a doctor's care, the patient decides not to take that medicine anymore or even visit the doctor. So, adverse events of medications make the patients more sensitive to the cost of medicine, and they wonder why they have to pay so much for a medicine that's causing them a certain complication."* [Informant 4]

### Disease-related factors

Determinants such as “type of disease,” “time of onset”, and “being under control” are disease-related factors that our informants stated:

*"When they have a persistent chronic illness, they might just get tired of taking medication. And if the medication is expensive, they may use that as an excuse not to take it. But they will probably be more willing to take medicine if their condition is a temporary or sudden illness."* [Informant 8]

or

*"What really rattles the older adult are acute conditions that occur suddenly. Patients are willing to pay more for acute diseases than for chronic ones. People who suffer from high blood pressure are often resistant to take their medications because they think they can handle it on their own or because the disease is chronic; they have to learn to live with it."* [Informant 6]

## Patient-related factors

This major theme refers to those factors originating from the traits of older adults, *e.g*., age, gender, education, etc.

In this regard, some of the participants pointed out as follows:

*"I feel that women are under more pressure because they don't pay."* [Informant 11]

or

*"Low level of education is a risk factor for poor adherence to treatment. Educated patients tend to adhere more closely to their prescribed medicine because they understand the importance of their therapy better. People who are not educated adequately know less about medication and science."* [Informant 9]

or

*"Patients with a strong sense of self-care will pay for their medication, even if it is expensive. "* [Informant 10]

## Discussion

To present a specific conceptualization of CRN with respect to the older adults with chronic diseases, an adapted model for Iranian patients was proposed. This adapted model depicts CRN to prescription medications as a dynamic and multifaceted phenomenon. In this regard, six categories of factors, including socio-economic, health system-related, healthcare provider-related, drug-related, disease-related, and patient-related factors, play a role as determinants of CRN. The proposed framework helps policymakers to focus on certain major themes and guides them to allocate resources in compliance with improving the older adult’s condition in the health system. Therefore, some effective interventions could be presented to the target group of older adults based on identified factors to mitigate the detrimental effects of CRN that are well-established by literature [29].

In line with previous studies, the results of our study included age, sex, and education as factors influencing CRN [22]. The results of our studies showed that the most important determinant is socio-economic factors. Among them, some cultural factors sensitize older adults to the cost of medications and increase the probability of CRN behavior. Previous studies confirm that social and cultural barriers could worsen CRN behavior among patients with different chronic diseases [30–33]. Reports indicated an association between social capital and CRN among older adults [11, 32]. Some other studies have emphasized the effects of employment status (unemployed, underemployed, employed), income, and access to non-employment-related resources (pensions, loans) on CRN [22]. However, since the target group in this study is older adults, the employment status could not be considered as a determinant.

Additionally, our results indicated that from the informants' point of view, external economic factors could lead to CRN among older adults patients by creating an inappropriate economic climate. Previous studies reported that the economic crisis may influence older adult’s ability to afford the cost of medication and decrease the level of medication adherence [34–36].

Regarding healthcare system-related factors, the most influential factor is the insurance coverage of older adult patients. Considering the importance of medication therapy in older adult patients with co-morbidities, there is a need to improve the affordability of medications by establishing a proper insurance system. In this regard, it was shown [7, 9, 37] that the administration of some costly anticancer medications followed by catastrophic expenditures might exaggerate CRN among older adult patients with no insurance coverage [6, 7]. In this context, the importance of access to and supervision of medicine use were emphasized in parallel with previous studies [13, 22].

Among healthcare provider-related factors, the communication between older adult patients and physicians, which is contributed to trust formation, has been frequently reported as a moderator of CRN behavior among patients [30, 31, 38]. Optimal time devoted to the older adults by a healthcare provider could create a constructive relationship between the older adults and the healthcare provider, followed by interpersonal trust and binding to the clinicians’ orders by patients [39]. This communication could focus on a conversation about the cost of medication and the economic status of older adult patients to estimate CRN. This may help older adults to cope with prescription costs and find alternative ways to overcome CRN [22]. Several studies have evidently documented the importance of cost conversation and its impact on the CRN behavior of patients [40–45].

Healthcare providers would also help reduce CRN among older adult patients by rationalizing medication therapy, specifically via managing polypharmacy [46]. Polypharmacy is a well-recognized issue for older adult patients who certainly struggle with multiple ailments [23, 47]. Multiple studies suggest that healthcare provider-patient relationships can greatly influence patients' decisions to take or to stop taking their medications. This relationship can be based on the doctor’s ability to provide appropriate information on dosage regimens, offer enough time to the patient, demonstrate empathy with the patient, and respect the patient’s concerns [22]. In addition, evidence is emerging that the relationships between healthcare providers and older adult patients are different from those of the patients in other age groups. This relationship is apparently sometimes more necessary due to cognitive and psychological features of the older adult patients [48].

Lee et al. (2018) cited that medication affordability as a drug-related factor is the key driver of the CRN [13]. This confirms our findings. In another study, it was revealed that patients are more likely to skip symptomatic medications given for temporary relief of less serious health problems (nonessential medications). Also, it was shown that the complexity of the dosage regimen and and profile of adverse drug reactions may affect patients’ response toward medication cost and adherence [22].

### Limitation

The strength of this study was to retrieve the opinions of informants from different settings of the healthcare system, which provided us with a comprehensive approach to the CRN topic by applying a systematic qualitative method for the first time in Iran. However, the first limitation of the current study is referred to not including the older adults’ opinions, as a key player of this scope. It was thought that interviews with older adult patients face inherent limitations that could not provide us with an in-depth information compared with key opinion leaders of the healthcare system.

The second limitation, like other qualitative studies, was the limited sample size. Furthermore, the informants were merely recruited from Tehran, the capital of Iran. These should be acknowledged when generalizing our findings to other communities. Despite these, the strength of this study was to retrieve the opinions of informants from different settings of the healthcare system, which provided us with a comprehensive approach to the CRN topic by applying a systematic qualitative method for the first time in Iran.

## Conclusion

Barriers to medication adherence are very complex, multifactorial in nature, and require much attention to be adequately addressed. Our results indicate that there are six major groups of factors affecting older adult's behavior toward CRN: socio-economic factors, health system-related factors, healthcare provider-related factors, drug-related factors, disease-related factors, and patient-related Factors. This framework will potentially enhance our understanding of the complex relationship between different barriers against medication adherence in older adult patients and may be used as a guide for policymakers and healthcare providers to design and implement effective initiatives. It is recommended that future studies use these findings and target multiple themes of barriers against the improvement of older adult patient’s health status.

## Supplementary Information

**Additional file 1.** COREQ (COnsolidated criteria for REporting Qualitative research) Checklist.

**Additional file 2.** Interview guide.

**Additional file 3.** Major themes, minor themes, and the corresponding exemplary quotes from informants.

## Data Availability

The data generated and analyzed during the current study are not publicly available due to privacy issues but are available from the corresponding author upon request.
