# Supplementary material for: An adapted model of cost-related medication nonadherence among older adult patients with chronic diseases: an Iranian qualitative study
Source: BMC Geriatr. 2023 Apr 1;23:208. doi: 10.1186/s12877-023-03907-0 (PMC10067279; doi:10.1186/s12877-023-03907-0)
Supplement: Supplementary file 2 — Additional file 2. Interview guide. [file 12877_2023_3907_MOESM2_ESM.docx]

*****Please note that in translating the following interviews from Persian into English, we have made a special effort to reproduce the conversational tone of the speakers as closely as possible. We have also tried to stay as close as possible to the original meaning of the speakers' words.

___________________________________________________________________

**Additional file 2. Interview guide**

| **Guiding Question** | **Check: Was that mentioned?** | **Definition and background** |
| --- | --- | --- |
| I would like to tell me about which factors can moderate CRN phenomenon among older patients with chronic disease in Iran? | - Socio-economic Factors - Health System-Related factors - Healthcare Provider-related Factors - Medication-related Factors - Disease-related Factors - Elderly-related Factors | CRN definition |
| What experiences have you had with elderly patients who showed CRN? |  | Definition of age limits in Iran (older than 60 years) |
