# Supplementary material for: An adapted model of cost-related medication nonadherence among older adult patients with chronic diseases: an Iranian qualitative study
Source: BMC Geriatr. 2023 Apr 1;23:208. doi: 10.1186/s12877-023-03907-0 (PMC10067279; doi:10.1186/s12877-023-03907-0)
Supplement: Supplementary file 3 — Additional file 3. Major themes, minor themes, and the corresponding exemplary quotes from informants. [file 12877_2023_3907_MOESM3_ESM.docx]

**Additional file 3. Major themes, minor themes, and the corresponding exemplary quotes from informants**

| **Major and Minor Themes** | **Main Category** | **Main quotes** |
| --- | --- | --- |
| Socio-economic Factors /Cultural Factors | Community attitudes towards physicians | "When there's a bad rap against doctors in the community, the older patients who distrust practitioners go and get their prescriptions, figure out the cost in their heads, do the math on paper, and say 'it's not clear at all what you the doctor said." |
|  | Social media education | "See, the thing is, even the English websites you search on over there, there is a lot of patient education about medications, their side effects, and the progression of the disease." |
|  | Educational programs for the elderly | "Through special training for the elderly, medication programs can be established. In our society, when someone retires or reaches old age, for example, they can get an educational brochure for their health so you can take your medications on time, ask your doctor for a comprehensive justification and ask the pharmacist." |
|  | Living in a nursing home | "Patients residing in a sanatorium can be the toughest to care. Many of these people feel unloved, which makes them feel hapless. We live for attention, but they don't understand how to give or receive attention. It doesn't matter from whom you get attention. It can be from your elderly roommate, but it doesn't happen in a nursing home. Sometimes the caregivers there get tired of their patients. " |
|  | Stigma | "Patients with milder psychotic disorders don't like the idea of coming to the hospital, getting drugged up, and possibly being branded for life. In cases involving elderly patients and psychiatric drugs, there's the potential for them to think their delusions are real and for their families to call them crazy. Heart disease is easy to diagnose, but their families call them crazy when they're on psychiatric meds. This is a major problem in society." |
|  | Elderly’s mental support | "Family mental support is also important. Elderly people with their kids around take their meds regularly and find it easier to get them." |
|  | Awareness and belief in medication therapy | "The knowledge and the things they get from others, from their community, or a peer, for example, about the availability of better medication, are more important, and generally the feedback they get from others is more important." |
| Socio-economic Factors /Economic Factors | The economic condition of the country | "Sometimes money is an issue. When the economy sucks and there aren't any jobs, that influences the patient's behavior. The patient's economic situation is important, but the country's economy is also important. Health influences the economy, and the economy influences health. But here, the economy as a whole affects people's medical behavior as much as any other." |
|  | Family purchasing power | "Unfortunately, there are some seniors who can't afford medicine. There are poor people in the region who can barely pay for their food, let alone their medicine. There are so many more basic needs that must be met." |
|  | Employment status | "Some people don't have a steady income or a permanent pension and have to get by with odd jobs. They can get (their medicines) at a certain time of the year and not at another." |
|  | Income level | "…their incomes are low, and they are living paycheck to paycheck. How will they afford that new medication?" |
|  | The financial burden imposed by family members | "The financial burden they get from others makes it hard for them to take care of themselves. They still have children at home that need to be provided for. These old people still have to work, for example, as cab drivers." |
|  | Family financial support | "But those intimate relationships with the families do help; they can alleviate some of the costs. Children can cover the costs. Those with special children who care for them usually don't have a problem. The child takes charge and says, 'Don't worry, I'll find a way to get it no matter what.' But those who have to shell out cash themselves have to squeeze things in their life." |
|  | Charity centers support | "The elderly need help paying for their medication through assistance centers or charity. Another option is that the elderly can get medical support. Instead of giving them cash, care centers prepare the medicines for the elderly and ensure they take medicine." |
| Health System-Related factors /Access | Medicine shortage | "Getting access to medicine can also be an issue. Some things can only be found in certain pharmacies in big cities, so if you don't live nearby, it might take a while to get there and back if you need to buy medicine." |
| Health System-Related factors /Insurance | Out-of-pocket costs | " I have seen elderly patients not care how much a certain medication costs. They don't buy medicine that isn't covered by insurance because they feel they're paying for it all by themselves." |
|  | Insurance coverage | " I have seen elderly patients not care how much a certain medication costs. They don't buy medicine that isn't covered by insurance because they feel they're paying for it all by themselves." |
| Health System-Related factors /Supervision and monitoring of medication therapy | - | " Health insurance should be connected with primary care physicians, who give elderly people their prescriptions. These physicians would ensure that patients follow their prescribed medicines and then send the drugs to their homes. Patients could get the medicine with two days left before the expiration date, and luckily, the system can track if their patients actually take the medicine. This can be heartwarming for the patient." |
| Health System-Related factors /Incentive policies for medication therapy | - | "Some services should be actively provided to the elderly. For example, we shouldn't say, 'Go and get medicine or do a test.' What if the patient doesn't follow? That's not an active health care system. In an active health care system, we need to follow up and offer services to the patient." |
| Healthcare Provider-related Factors / Healthcare-provider interaction | Devoted time for the visit | "Doctors usually don't take the time to tell patients how to take their medications. Doctor visits mean that doctors don't tell patients about the side effects or how much to take. This leads to patients forgetting to take their meds." |
|  | Communication with the elderly | "… there is no communication between the doctor and the patients. In other words, in the communication between doctor and patient, the more information the patient has, the more they will want to get a prescription from the doctor. If you have a good relationship with your doctor, you will listen to what they say and do it." |
|  | Talking about the importance of taking medicine | "My opinion is that it depends on how much the doctor can influence patients and convince them to take the treatment. I think that in the field of the elderly, doctors should have the ability to convince the patient is part of the treatment." |
| Healthcare Provider-related Factors / Medication treatment management | Polypharmacy management | "Reducing the number of medications, a patient takes effectively saves money. Sometimes a patient has already been prescribed medication. But as the patient grows older, that medication is no longer needed. Over time, it should be discontinued in people with diabetes who develop nephropathy and whose insulin lifespan is longer. Imagine a person with diabetes whose sleep is a wreck and who gets neuropathic pain. Gabapentin helps, lowering the need for extra medications. The more we can use it and other meds like it, the higher the adherence and the lower the cost." |
|  | Medication reconciliation | "Just as we say that the elderly should bring all their medicines. All specialists should have a comprehensive view. It should not be that the heart specialist only gives their own medicine, which then interferes with each other. The cost for the elderly will also increase. One thing is for the doctors to check all the medicines and prescribe the medicine necessary for the elderly." |
|  | Teaching about medication efficacy | "So if I don't explain to the patient that this medicine I give does not solve your problem exactly, but it prevents the progress of the disease if I don't give this training to the patient, it prevents the patient's adherence." |
|  | Management of medication side effects | "Doctor visits mean that doctors don't tell patients about the side effects or how much to take" |
| Healthcare Provider-related Factors / Physicians’ awareness of the economic situation of the elderly | - | "When we want to prescribe medication for a patient, we first ask: Where do you work? Where do you live? Sometimes we even ask about the house, whether it's rented or owned by you. That's a ballpark figure. We consider the patient's financial situation, that is, the extent of what they can afford, and then we prescribe medications accordingly. And if I don't say there's a generic alternative, they won't get it, and it will be as if they never came in because the case will be closed, and they won't get treatment. It's important to pay attention to the patient's finances and prescribe accordingly." |
| Healthcare Provider-related Factors / Physician awareness of the medication costs | - | "I'm a Pharmacotherapist, so this is a very high priority. I discuss this (prescription cost) with the patient. If I see that they can't afford it, I give them an alternative or find an alternative medication." |
| Emphasis on brand medication | - | "The doctor and the patient or their accompanying family member/friend do not communicate properly. As a result, Doctors don't clearly instruct their patients that the brand-name medicine they prescribe is no better than the generic one. The doctor prescribes the brand-name drug. The patient cannot afford to buy the drug whether it has no means to pay for it or the pharmacy has no stock of it." |
| Medication-related Factors / Efficacy | - | "If domestic pharmaceutical companies offer better-quality drugs, this problem will be largely avoided. Ultimately, the desired results will be achieved if generics are of good quality and covered by insurance." |
| Medication-related Factors / Dosage form | - | "When I order sustained release formulation for older patients, and I explained that it's expensive, but it has not some adverse events, and it makes you better, then he/she is convinced." |
| Medication-related Factors /Price | - | "Often, I'd find them (the elderly) asking about the price of the medication and leaving out the most expensive one, which is usually the main drug. Without it, the illness could get worse. At that time, I'd instruct them never to leave this out. Most of them wouldn't accept what I say." |
| Medication-related Factors / Side effects | - | "For instance, because of a medicine side effect or even a complication caused by the illness while under a doctor's care, the patient says, 'I am not taking that medicine anymore or even seeing that doctor.' That makes the patients more sensitive to the cost of medicine, and they wonder why they have to pay so much for medicine causing them a certain complication." |
| Medication-related Factors / Brand or generic | - | "Doctors may prescribe some brand medicines for their patients. But those patients are quite willing to take a lower dose of the medicines in order to reduce their drug costs. They believe that brand-name drugs are more effective and are not afraid to try a lower dose. Some patients take only half of their pills or take them every other day instead of all or every day." |
| Medication-related Factors / Onset of action | - | "That's especially the case when it's a medication that doesn't provide immediate relief and makes you feel even more miserable immediately after taking it. Many patients with medications to lower their blood pressure don't use them as prescribed because they don't see any immediate change in their symptoms. So they rely on pain pills, so they don't have to feel lousy." |
| Disease-related Factors / Being under control | - | "When they have a persistent chronic illness, they might get tired of taking medication. And if the medication is expensive, they may use that as an excuse not to take it. But they will probably be more willing to take medicine if it's a temporary or sudden illness." |
| Disease-related Factors / Type of disease | - | "People pay for medicines when they're sick with cancer; they're terrified of it; they'd even literally go 'sell the carpet under their feet just to be well. Kids must work hard; they accept it if the sickness is deadly and has a bad rep." |
| Disease-related Factors / Time of onset of the disease | - | "Chronic diseases or diseases like diabetes do not have severe symptoms but have high prevalence and hurt massively in the long run. Therefore, whether chronic or not does not make much difference to the patient." |
| Elderly-related Factors  / Health literacy |  | "Low literacy is a risk factor for poor adherence to treatment. Educated patients tend to adhere more closely to their prescribed medicine because they understand their therapy better. People who don't have a good education know less about medicine and doctors, so they don't really believe in them. So when you talk to them, some people are convinced, but not others." |
| Elderly-related Factors  / Elderly attitude to brand medicine |  | "For example, if their doctor prescribes a brand medicine, instead of generic medicine, they are willing to take a brand medicine even with a lower dose so that the cost does not increase. Because they believe more in the effectiveness of the brand type. I have seen many examples of dose reduction, people who take half their pills instead of the whole pill, or take it every other day instead of every day." |
| Elderly-related Factors  / Addiction |  | "Older people with addiction have less self-care or vice versa. So it is more likely that an older person who has an addiction will show CRN behavior. " |
| Elderly-related Factors  / The level of self-care |  | "Patients with a strong sense of self-care will pay for their medication, even if it is expensive. This is also generally true about patients having chemotherapy. They'd go so far as to literally 'sell the carpet under their feet.'" Of course, some contradictory behavior can also be observed." |
| Elderly-related Factors  / Physical ability | Dependency on others to supply or consume medicines | "Some of it has to do with the patient's physical limitations. A patient may not be able to use their fingers properly to open the medication bottle, or they may not be able to inject themselves. Also, visual issues can play a part." |
| Elderly-related Factors  / Perception of the severity of the disease | - | "A small heart problem that has no outcomes on their health will steal their hearts away, but blood pressure is often taken for granted." |
| Elderly-related Factors  / Educational level | - | "But educated people who are more health-conscious are more cautious and even ask, 'What should I do now that I've missed a dose?'' |
| Elderly-related Factors  / Underlying diseases | Depression | "For example, a person with depression usually does not take their medicine, which may be due to poverty or lack of willingness to receive medical treatment." |
|  | Cognitive disorders | "This problem occurs more often in the elderly who have dementia." |
| Elderly-related Factors  / Gender | - | "I think women are under more pressure for pay because most of them are dependent to the others e.g., their husband, for medical costs." |
| Elderly-related Factors  / Age | - | "When they get older, they refuse to take their medication: 'I'm not going to be around much longer; it doesn't make sense to waste all this money; I'll leave it to the funeral home.'" |
